# Presence of Trace Metals in the Biological Samples of Prostate Cancer Patients: A Systematic Review of Case-Control Studies

**DOI:** 10.3390/cancers18020236

**Published:** 2026-01-13

**Authors:** Unathi A. Tshoni, Thokozani P. Mbonane, Phoka C. Rathebe

**Affiliations:** Department of Environmental Health, Faculty of Health Sciences, Doornfontein Campus, University of Johannesburg, P.O. Box 524, Johannesburg 2006, South Africa; tshoniunathi@gmail.com (U.A.T.); tmbonane@uj.ac.za (T.P.M.)

**Keywords:** prostate cancer, trace metals, cancer risk, biological samples

## Abstract

**Simple Summary:**

Studies on trace metals and prostate cancer are examined in this review. It was discovered that, in comparison to healthy males, men with prostate cancer frequently have greater levels of cadmium (Cd) and lower levels of zinc (Zn) and selenium (Se). Lead (Pb), nickel (Ni), arsenic (As), and mercury (Hg) did not exhibit obvious connections. Overall, the results point to a possible link between prostate cancer and cadmium exposure, as well as zinc and selenium deficits; however, further research is required to address study limitations and other affecting factors.

**Abstract:**

**Background/Objective:** Prostate cancer (PCa) is the leading cause of death in the ageing male population across the globe, and trace metals have garnered much attention due to their sometimes-dual role in cellular mechanisms, as such contribute to the development and progression of prostate cancer. **Methods:** This review consolidates the results of case-control studies that investigated the concentrations of certain trace metals—Arsenic (As), cadmium (Cd), mercury (Hg), manganese (Mn), nickel (Ni), lead (Pb), selenium (Se), and zinc (Zn) in various biological samples. **Results:** There are decreased concentrations of Se and Zn and increased Cd concentrations in samples of PCa patients when compared to healthy controls. As, Hg, Ni, and Pb concentrations have proven to be insignificant. **Conclusions:** There are other variables to consider and limitations that need to be investigated in studies of this nature; however, the results have been consistent in that increased exposure to toxic metals such as Cd, along with a deficiency in protective essential nutrients like Zn and Se, tends to produce a prostatic environment.

## 1. Introduction

One of the leading causes of cancer-related deaths for men is prostate cancer, with a steady increase in global burden because of ageing populations [1,2]. Even though age, genetic predisposition and lifestyle factors have played a huge role, in recent years, research has indicated that excessive exposure to environmental factors such as trace metals also plays a pivotal role in the development of prostate cancer [3,4,5]. With continued industrialisation and environmental pollution, the levels of trace metals are increasing in food and water sources, as well as air, thus influencing cancer risk [6,7].

Whilst trace metals like zinc, selenium, and manganese are useful for certain physiological functions, they are also known to be disruptive to certain cellular mechanisms when in abundance in the body [8,9,10]. On the other hand, trace metals, such as lead and cadmium, with no known physiological importance, have also been implicated in cancer risk [11,12]. The significance of these trace metals in cancer research is made apparent by their impact on cellular proliferation, inflammation, and oxidative stress [13,14]. Recently, a review by Tshoni et al. [15] indicated that trace metals have an essential role in the risk of developing prostate cancer, acting as a safeguard and a possible danger; thus, highlighting how crucial it is to manage trace metal exposure in a balanced manner.

Over the past decade, several case-control experimental studies have emerged, examining the presence of trace metals in biological samples from patients diagnosed with prostate cancer in an effort to identify significant variations in the concentration of trace metals between cancer-diagnosed individuals and healthy control groups [16,17,18,19]. These studies used an array of analytical methods: atomic absorption spectroscopy (AAS), inductively coupled plasma mass spectrometry (ICP-MS), inductively coupled plasma atomic emission spectroscopy (ICP-AES), particle-induced X-ray emission (PIXE), and X-ray fluorescence (XRF), as they are known for accurate detection and highly accurate quantification of trace metal concentrations.

This review intends to consolidate the results from case-control studies that investigated the concentrations of trace metals in various biological samples—blood, urine, hair, nails, and prostatic tissue. It seeks to broaden our understanding of the mechanisms by which trace metals impact prostate cancer pathophysiology. The data could offer significant insight into risk assessment and early detection.

## 2. Search Strategy

This review followed the PRISMA guidelines [20]. The review has been registered on the International Prospective Register of Systematic Reviews platform and is awaiting publication and registration (ID: 1143873). The data included were gathered by thoroughly searching scientific databases for observational studies [case controls] on the subject matter (Supplementary S1). The databases used were PubMed and Science Direct, restricted to English. The search keywords were as follows: “trace metals” AND “prostate cancer patients”, “trace metals” AND “prostate cancer”, “observational study” AND “biological samples” AND “prostate cancer”, and “serum trace elements” AND “prostate cancer risk. The search was limited to peer-reviewed case-control publications published between January 2000 and December 2024, with clearly defined protocols, control groups and employed trusted analytical methodologies [AAS, ICP-MS, PIXE and XRF] to measure trace metals in biological samples of prostate cancer patients. Studies that lacked quantitative trace metal analysis data, control groups or defined study designs, animal models, reviews, and meta-analyses were excluded.

### 2.1. Study Selection and Data Collection

For studies to be included in this review, they had to meet the PECO criteria: (1) the study population (P) was restricted to individuals with an official prostate cancer diagnosis; (2) exposure (E) to trace metals as proven by biological samples; a specified comparator (C), including individuals without cancer; and the [4]outcome (O) was the overall incidence of prostate cancer [21]. There was no comprehensive search for grey literature sources; only articles from peer-reviewed databases were used to reduce publication bias. For risk of bias, the Newcastle–Ottawa Scale (NOS) [22] was used (Table 1) by assigning stars based on three categories—selection, comparability and outcome. Subsequently, all the included studies were published in the English language and only had human participants. Only the original research articles (case-control) were used to provide the empirical data and were considered for analysis in this review article. Meta-analyses and systematic reviews were not included in the analysis and were only used solely to provide context or support for the findings.

### 2.2. Data Extraction and Analysis

For each relevant study, the following information was extracted and summarised in a table format: the study population, sample size, biological sample analysed, trace metals, and key findings on trace metal concentrations in the biological samples of prostate cancer patients and controls. Two reviewers separately used a piloted screening form to conduct full-text eligibility assessment and title/abstract screening. Two reviewers also independently carried out data extraction in duplicate using a piloted extraction template that recorded study identifiers, design, population, sample type, assay method, metal(s) measured, units, concentrations or effect estimates (with measures of variance), NOS score, covariates adjusted for, and any mechanistic endpoints. The principal effect measure for each study was documented along with the conversions or other methods utilised to produce it. Discrepancies between the reviewers were resolved by discussion and consensus; unresolved disagreements were adjudicated by a third reviewer.

The data were then studied for trends and potential mechanisms linking prostate cancer risk and trace metal exposure by extracting data, such as study designs and population traits, looking for patterns across studies and using evidence as discussed in studies to deduce roles of each trace metal (Table 2, Table 3, Table 4 and Table 5).

The results did not undergo systematic meta-analysis because the studies were varied in terms of biological matrices (serum, plasma, tissue, urine), exposure metrics (various units and reporting formats), and completeness of provided variance data (SD/SE).

## 3. Results

**Figure 1:**
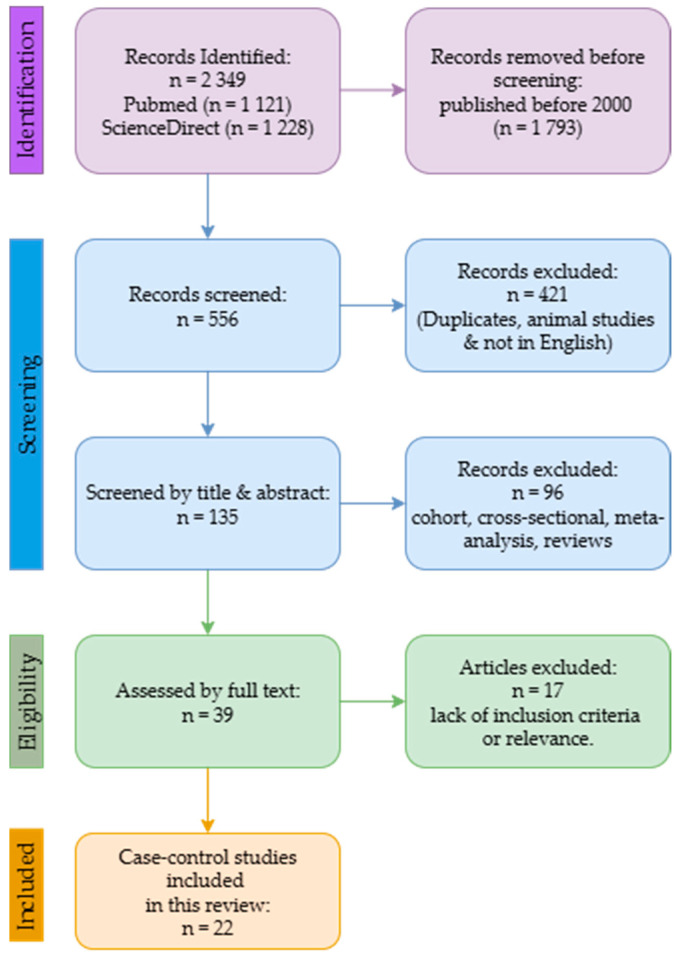
Schematic overview of the review based on the PRISMA guidelines.

This review made use of the PRISMA guidelines (Figure 1) to select articles, with respect to the inclusion and exclusion criteria. Through the systematic search, 2349 articles were retrieved, of which 1121 were obtained from PubMed and 1228 from the ScienceDirect database. After removing 1793 articles published before 2000, 556 articles were screened, and 421 were duplicates, animal studies, not in English, and on different cancers. The remaining 135 articles were screened by title and abstract. This step resulted in the exclusion of 96 studies [cohorts, cross-sectional, reviews, and meta-analyses]. Then, 39 full texts were subjected to additional checks to assess the criteria and quality assessment. A further 17 studies were excluded due to a lack of inclusion criteria or relevance. This current review includes the 22 articles.

This review used the Newcastle–Ottawa Scale to assess bias (Table 1), and 22 case-control studies were assessed. Of the twenty-two studies, only one study had moderate quality (score = 6), suggesting a possibility of bias, albeit minimised, which is associated with confounding variables. The other 21 studies had a low to no risk of bias (score > 7), thus diminishing information bias and improving study confidence. This aspect of scientific integrity eliminates the likelihood of exaggerating the relationship between prostate cancer risk and trace metals.

Table 2 shows a comprehensive overview of the descriptive characteristics of the included case-control studies. These studies are geographically diverse: with five having been conducted in Nigeria [16,23,24,25,26]; four in Turkey [27,28,29,30]; three in Saudi Arabia [18,19,31], as well a single study each in India [32], Italy [33], Malaysia [34], Germany [35], Poland [36], Australia [37], Singapore [17], Taiwan [38], Russia [39], and Pakistan [40].

**Table 2:** Descriptive characteristics of the case-control studies included in this review.

Study	Country	Trace Metals	Biological Sample	Study Group [*n*]	Control Group [*n*]	Analytical Method
[Bede-Ojimadu et al., 2023] [16]	Nigeria	Zn, Cd	Blood, urine	82	98	ICP-MS
[Lim et al., 2019] [17]	Singapore	Mn, Zn, As, Se, Cd, Pb, Ni	Serum	141	114	ICP-MS
[Saleh et al., 2017] [18]	Saudi Arabia	Zn, Mn, Se	Hair	58	52	ICP-MS
[Saleh et al., 2020] [19]	Saudi Arabia	Mn, Zn, Se	Serum	40	30	ICP-MS
[Adedapo et al., 2012] [23]	Nigeria	Se, Zn, Mn	Serum	40	40	AAS
[Amadi & Aleme, 2019] [24]	Nigeria	Zn	Serum	220	220	AAS
[Olooto et al., 2021] [25]	Nigeria	Se, Zn	Serum	55	25	AAS
[Onyema-iloh et al., 2014] [26]	Nigeria	Zn, Se	Serum	100	50	AAS
[Karimi et al., 2012] [34]	Malaysia	Se, Zn, Mn	Hair, nails	50	50	ICP-MS
[Aydin et al., 2006] [27]	Turkey	Zn	Serum	25	24	AAS
[Eken et al., 2016] [28]	Turkey	Zn, Mn, Se	Serum	42	40	AAS
[Kaba et al., 2014] [29]	Turkey	Mn, Zn, Pb, Cd	Serum	30	32	AAS
[Ozmen et al., 2006] [30]	Turkey	Ni, Zn	Blood	20	21	AAS
[Saleh et al., 2019] [31]	Saudi Arabia	Zn, Se, Cd	Serum	58	30	ICP-MS
[Guntupalli et al., 2007] [32]	India	Mn, Ni, Zn, Se	Tissue	27	27	PIXE
[Vinceti et al., 2007] [33]	Italy	Cd	Toenails	40	58	AAS
[Steinbrecher et al., 2010] [35]	Germany	Se	Serum	248	492	DRC-ICP-MS
[Drozdz-Afelt et al., 2024] [36]	Poland	Ni, As, Cd, Pb, Hg, Zn	Blood	66	64	ICP-MS
[Dhillon et al., 2022] [37]	Australia	Se, Zn	Plasma	116	132	ICP-AES
[Chang et al., 2018] [38]	Taiwan	Cd, Ni, Hg, Pb, Zn, As	Serum	20	23	ICP-MS
[Zaichick & Zaichick, 2019] [39]	Russia	Zn	Prostatic fluid	24	38	XRF
[Qayyum & Shah, 2014] [40]	Pakistan	Cd, Mn, Ni, Pb, Zn	Blood, Hair, Nails	B-74 H-67 N-60	66 67 60	AAS

AAS—Atomic Absorption Spectroscopy, ICP-MS—Inductively Coupled Plasma Mass Spectrometry, XRF—X-ray Fluorescence, PIXE—Particle-induced X-ray emission, ICP-AES—Inductively Coupled Plasma Atomic Emission Spectroscopy.

Within these 22 studies, the focus was on various trace metals (Table 3, Table 4 and Table 5)—20 focused on zinc, 12 on selenium, 5 on lead, 9 on manganese, 8 on cadmium, 6 on nickel, 3 on arsenic, and 2 on mercury. These also varied in the biological samples being investigated—serum > blood/plasma > hair = nails > tissue = prostatic fluid. The two most often used techniques in this research are AAS and ICP-MS, each of which has been used in ten studies. XRF and PIXE, on the other hand, have only been used in one study each.

**Table 3:** Descriptive characteristics and concentrations (µg/L) of the case-control studies included in this review for fluid and tissue biological samples.

Trace Metal	*p*-Value	Study Group—Concentration Mean ± Standard Dev (Min–Max)	Control Group—Concentration Mean ± Standard Dev (Min–Max)	Age (Years) Mean ± SD/Range Case (Control)	Country/Region	References
**Blood/Serum/Plasma**						
**Zinc**	0.666	4118.57 (3460.31–4827.80)	4071.10 (3554.53–4639.60)	64–76 (62–75)	Nigeria	[16]
	1.72 × 10^−15^	857.4 ± 162.1 (441.1–1557.8)	690.7 ± 149.3 (373.9–1129.7)	≥50	Singapore	[17]
	<0.05	0.51 ± 0.09 ^a^	0.87 ± 0.29 ^a^	68.2 ± 5.2 (65.8 ± 6.8)	Saudi Arabia	[19]
	0.107	119,800.0 ± 25,200.0	114,400.0 ± 25,200.0	68.5 ± 9.6 (65.2 ± 6.7)	Nigeria	[23]
	<0.001	1064.46 ± 341.62 (384.61–2013.54) ^a^	1722.82 ± 505.65 (463.79–2748.82) ^a^	69.7 ± 7.7 (68.97 ± 7.3)	Nigeria	[24]
	0.000	43.3 ± 3.3	139.9 ± 7.7	NS *	Nigeria	[25]
	<0.05	1687.80 ± 598.00	1477.50 ± 420.50	NS *	Nigeria	[26]
	<0.01	5760.0 ± 102.0	7110.0 ± 164.0	67.5 ± 8.8 (65.0 ± 6.0)	Turkey	[27]
	<0.05	0.86 ± 0.22 ^a^	0.91 ± 0.18 ^a^	46–85	Turkey	[28]
	0.001	712.72 ± 339.29 (67.09–1173.0)	2945.33 ± 498.18 (2125.0–4021.0)	65.4 ± 4.2 (62.8 ± 5.8)	Turkey	[29]
	<0.001	4375.0 ± 620.0	5843.0 ± 747.0	72.5 ± 7.8 (66.3 ± 8.3)	Turkey	[30]
	0.001	0.73 ± 0.41	2.64 ± 0.56	71.1 ± 5.4	Saudi Arabia	[31]
	0.003	4735.6 ± 1124.3 (3005.3–12,017.9)	5211.3 ± 1463.0 (2692.3–12,539.8)	≥48 (>50)	Poland	[36]
	0.23	0.77 ± 0.1 (0.52–1.24)	0.78 ± 0.01 (0.52–1.04)	71.2 ± 7.18(69.1 ± 7.99)	Australia	[37]
	0.001	421.9 ± 132.2	600.26 ± 189.01	73.4 ± 6.7 (71.3 ± 8.1)	Taiwan	[38]
	<0.05	2.483 ± 0.210 * (0.059–8.536)	6.571 ± 0.656 * (0.940–32.74)	32–75 (27–68)	Pakistan	[40]
**Cadmium**	0.000	1.30 (0.003–1.88)	0.003 (0.003–0.003)	64–76 (62–75)	Nigeria	[16]
	0.82	0.22 ± 0.041 (0.22–0.58)	0.25 ± 0.32 (0.22–3.56)	≥50	Singapore	[17]
	0.630	1.32 ± 0.15 (2.01–9.54)	1.11 ± 0.11 (1.01–1.35)	65.4 ± 4.2 (62.8 ± 5.8)	Turkey	[29]
	0.63	0.0015 ± 0.001	0.0011 ± 0.001	71.1 ± 5.4	Saudi Arabia	[31]
	0.002	0.46 ± 0.45	0.9 ± 0.35	73.4 ± 6.7 (71.3 ± 8.1)	Taiwan	[38]
	<0.05	1.084 ± 0.107 * (0.059–3.832)	0.774 ± 0.096 * (0.017–3.946)	32–75 (27–68)	Pakistan	[40]
**Selenium**	4.91 × 10^−10^	131.4 ± 30.2 (86.1–296.9)	108.9 ± 27.0 (55.7–220.0)	≥50	Singapore	[17]
	<0.005	0.07 ± 0.01 ^a^	0.14 ± 0.03 ^a^	68.2 ± 5.2 (65.8 ± 6.8)	Saudi Arabia	[19]
	0.741	586.0 ± 128.0	597.0 ± 123.0	68.5 ± 9.6 (65.2 ± 6.7)	Nigeria	[23]
	0.000	615.0 ± 34.4	956.2 ± 13.9	NS *	Nigeria	[25]
	<0.05	43.00 ± 14.52	82.092 ± 7.26	NS *	Nigeria	[26]
	<0.05	68.52 ± 6.93 ^a^	83.49 ± 5.69 ^a^	46–85	Turkey	[28]
	0.001	53.00 ± 25.00	193.00 ± 32.00	71.1 ± 5.4	Saudi Arabia	[31]
	NS *	86.2 ± 14.2	87.7 ± 13.4	58.1 ± 4.8 (58.1 ± 4.8)	Germany	[35]
	0.002	116.11.59 (71.83–157.6)	125.6 ± 2.56 (79.17–238.1)	71.2 ± 7.18(69.1 ± 7.99)	Australia	[37]
**Manganese**	6.05 × 10^−7^	5.84 ± 12.6 (0.50–131.2)	2.24 ± 3.69 (0.50–29.0)	≥50	Singapore	[17]
	<0.005	0.01 ± 0.001 ^a^	0.0026 ± 0.001 ^a^	68.2 ± 5.2 (65.8 ± 6.8)	Saudi Arabia	[19]
	0.175	36,800.0 ± 5800.0	37,100.0 ± 7900.0	68.5 ± 9.6 (65.2 ± 6.7)	Nigeria	[23]
	<0.05	0.62 ± 0.19 ^a^	0.22 ± 0.08 ^a^	46–85	Turkey	[28]
	0.001	97.53 ± 75.35 (1.10–437.80)	746.06 ± 219.83 (111.14–989.91)	65.4 ± 4.2 (62.8 ± 5.8)	Turkey	[29]
	<0.05	1.524 ± 0.140 * (0.93–5.761)	0.895 ± 0.068 * (0.032–2.066)	32–75 (27–68)	Pakistan	[40]
**Arsenic**	1.89 × 10^−9^	4.78 ± 4.46 (0.50–26.4)	2.66 ± 3.67 (0.50–25.0)	≥50	Singapore	[17]
	<0.001	0.9 ± 1.2 (0.0–6.4)	0.3 ± 0.5 (0.0–2.7)	≥48 (>50)	Poland	[36]
	0.078	6.8 ± 7.2	13.14 ± 8.66	73.4 ± 6.7 (71.3 ± 8.1)	Taiwan	[38]
**Lead**	0.00024	5.26 ± 42.6 (0.60–505.6)	1.31 ± 6.82 (0.60–73.4)	≥50	Singapore	[17]
	0.001	57.96 ± 30.62 (27.41–169.60)	1.21 ± 0.13 (1.0–1.5)	65.4 ± 4.2 (62.8 ± 5.8)	Turkey	[29]
	0.001	17.4 ± 61.9 (0.0–508.9)	31.9 ± 152.4 (0.0–1231.1)	≥48 (>50)	Poland	[36]
	0.015	18.5 ± 17	54.6 ± 62.04	73.4 ± 6.7 (71.3 ± 8.1)	Taiwan	[38]
	<0.05	3.658 ± 0.358 * (0.023–12.67)	2.248 ± 0.216 * (0.019–7.474)	32–75 (27–68)	Pakistan	[40]
**Nickel**	<2.2 × 10^−16^	17.8 ± 41.6 (1.46–438.0)	2.10 ± 2.27 (1.46–17.3)	≥50	Singapore	[17]
	<0001	426.00 ± 84.00	238.00 ± 101.00	72.5 ± 7.8 (66.3 ± 8.3)	Turkey	[30]
	0.295	30.9 ± 48.7 (0.0–234.7)	21.1 ± 38.7 (0.0–239.0)	≥48 (>50)	Poland	[36]
	<0.001	57.1 ± 22.9	54.71 ± 35.43	73.4 ± 6.7 (71.3 ± 8.1)	Taiwan	[38]
	<0.05	4.335 ± 0.384 * (0.030–10.35)	2.687 ± 0.241 * (0.012–8.836)	32–75 (27–68)	Pakistan	[40]
**Mercury**	0.808	1.15 ± 1.25 (0.07–7.54)	1.05 ± 0.77 (0.12–3.62)	≥48 (>50)	Poland	[36]
	<0.001	6.9 ± 9	1.79 ± 1.7	73.4 ± 6.7 (71.3 ± 8.1)	Taiwan	[38]
**Urine**						
**Zinc**	0.234	638.87 (527.18–774.57)	696.95 (591.96–820.36)	64–76 (62–75)	Nigeria	[16]
**Cadmium**	0.221	1.65 (1.33–2.042)	1.95 (1.64–2.33)	64–76 (62–75)	Nigeria	[16]
**Tissue**						
**Zinc**	8.0 × 10^−6^	50.7 ± 3.26	366.7 ± 5.59	52.5 ± 9.2	India	[32]
**Manganese**	0.059	11 ± 1.82	3 ± 1.45	52.5 ± 9.2	India	[32]
**Selenium**	0.0059	1.1 ± 0.34	3.2 ± 0.41	52.5 ± 9.2	India	[32]
**Nickel**	0.0006	24.32 ± 2.95	2.4 ± 0.72	52.5 ± 9.2	India	[32]
**Prostatic fluid [mg/L]**						
**Zinc**	0.64	62.0 ± 98.3	598 ± 207	65 ± 10 (59 ± 11)	Russia	[39]

* Standard error [SE]; ^a^ µg/mL; NS—Not Specified.

**Table 4:** Descriptive characteristics and concentrations (µg/g) of the case-control studies included in this review for keratinised biological samples.

Trace Metal	*p*-Value	Study Group—Concentration Mean ± Standard Dev (Min–Max)	Control Group—Concentration Mean ± Standard Dev (Min–Max)	Age (Years) Mean ± SD/Range Case (Control)	Country/Region	References
**Hair**						
**Zinc**	<0.005	3.1 ± 0.7	4.4 ± 0.4	71.1 ± 5.4 (66.8 ± 7.8)	Saudi Arabia	[18]
	0.018	3.29 ± 2.22 (1.48–3.99)	4.29 ± 2.53 (0.05–0.08)	72.4 ± 6.7 (71.7 ± 7.9)	Malaysia	[34]
	<0.05	160.7 ± 9.07 * (59.75–591.0)	582.7 ± 25.73 * (6.85–1207)	38–72 (31–68)	Pakistan	[40]
**Cadmium**	<0.05	1.629 ± 0.159 * (0.05–4.70)	1.021 ± 0.090 * (0.05–2.80)	38–72 (31–68)	Pakistan	[40]
**Selenium**	<0.005	7.3 ± 1.4	11.5 ± 2.1	71.1 ± 5.4 (66.8 ± 7.8)	Saudi Arabia	[18]
	0.001	7.15 ± 3.5 (4.48–9.74)	10.4 ± 4.52 (6.88–14.00)	72.4 ± 6.7 (71.7 ± 7.9)	Malaysia	[34]
**Manganese**	<0.005	0.083 ± 0.02	0.058 ± 0.03	71.1 ± 5.4 (66.8 ± 7.8)	Saudi Arabia	[18]
	0.001	0.07 ± 0.04 (0.05–0.09)	0.055 ± 0.05 (0.01–0.10)	72.4 ± 6.7 (71.7 ± 7.9)	Malaysia	[34]
	<0.05	4.65 ± 0.37 * (0.40–12.70)	2.85 ± 0.24 * (0.15–7.50)	38–72 (31–68)	Pakistan	[40]
**Nickel**	<0.05	37.21 ± 2.82 * (1.05–85.95)	19.35 ± 1.84 * (0.05–57.25)	38–72 (31–68)	Pakistan	[40]
**Lead**	<0.05	35.87 ± 2.49 * (0.75–82.00)	13.03 ± 1.41 * (0.2549.35)	38–72 (31–68)	Pakistan	[40]
**Nails/Toenails**						
**Zinc**	0.01	2.70 ± 1.49 (1.31–4.07)	3.97 ± 4.06 (2.73–4.36)	72.4 ± 6.7 (71.7 ± 7.9)	Malaysia	[34]
	NS	114.2 ± 4.53 * (22.5–182.1)	122.7 ± 8.54 * (45.00–383.2)	32–75 (27–68)	Pakistan	[40]
**Selenium**	0.001	7.23 ± 3.11 (5.26–8.98)	9.03 ± 3.69 (5.90–11.69)	72.4 ± 6.7 (71.7 ± 7.9)	Malaysia	[34]
**Manganese**	0.001	0.10 ± 0.06 (0.06–0.13)	0.05 ± 0.04 (0.01–0.08)	72.4 ± 6.7 (71.7 ± 7.9)	Malaysia	[34]
	<0.05	11.98 ± 1.05 * (0.25–35.42)	8.63 ± 0.75 * (1.25–24.58)	32–75 (27–68)	Pakistan	[40]
**Cadmium**	<0.05	5.66 ± 0.50 * (0.33–14.75)	3.06 ± 0.29 * (0.33–8.29)	32–75 (27–68)	Pakistan	[40]
	0.004	4.7 (1.3–17.5)	0.088 (0.018–0.210)	43–83	Italy	[33]
**Lead**	NS	24.49 ± 2.17 * (2.00–67.00)	23.83 ± 2.53 * (1.30–67.92)	32–75 (27–68)	Pakistan	[40]
**Nickel**	<0.05	101.1 ± 10.19 * (0.85–267.1)	56.65 ± 5.36 * (1.88–153.2)	32–75 (27–68)	Pakistan	[40]

* Standard error [SE]; NS—not significant.

**Table 5:** Summary of the general comparative concentrations of trace metals in samples of prostate cancer cases and matched controls.

Trace Metal	Cases vs. Controls	Analysis
Zn	↓ decreased in cases	Linked to impaired antioxidant and DNA damage
Se	↓ decreased in cases	Linked to oxidative damage
Mn	↑ slightly increased in cases	Variable by exposure source
Cd	↑ increased in cases	Suggests toxic burden
Pb	↑ slightly increased in cases	Possible environmental contribution
Ni	↑ increased in cases	Possible environmental contribution
As	↑ slightly increased in cases	Possible environmental contribution
Hg	↑ slightly increased in cases	Possible environmental contribution

↑ increase; ↓ decrease.

Across the included studies, cadmium was the most consistently elevated trace metal in PCa cases. Of the seven studies examining cadmium in blood/plasma samples, four (57%) of them reported significantly higher levels in cases compared to controls; the remaining 43% found no significant differences, even though lower levels were reported for cases. Only one study reported on levels of cadmium in urine; other cases had higher levels than controls, but it was not significant. For hair, the one study that reported on cadmium showed significantly higher levels compared to controls. Both studies reporting on cadmium in nails/toenails had higher levels of cadmium in cases compared to controls, but only one was significantly higher (50%).

For zinc, the blood concentration results were more diverse; of the 16 studies examining zinc in blood samples, 10 (62%) were statistically significant, with cases having lower zinc compared to controls, and 3 (19%) being statistically significant but with zinc higher in controls compared to cases. The remaining three (19%) did not have any significance. For urine, only one study reported on zinc, and it did not report any significant results. All three (100%) studies that reported zinc in hair samples showed it to be significantly lower in cases compared to controls. Only one of the two (50%) studies reporting on zinc in nail material showed significance. One study also reported on zinc in prostatic fluid; although the results were not significant, the concentrations were lower in cases compared to controls.

Selenium was assessed in nine studies for blood samples, with eight of the studies showing lower levels in cases compared to controls, but only six (67%) being statistically significant, one (11%) showing higher and statistically significant levels in controls compared to cases. The remaining studies (22%) showed no significant levels when compared to their counterparts. Selenium in hair samples was reported by two studies, and both studies showed selenium concentrations to be significantly lower in cases compared to controls, and the one study that reported on nail samples also showed significantly lower levels in cases compared to controls.

Six studies reported on blood manganese, and four (67%) reported significantly higher levels in cases compared to controls, and the remaining two (33%) reported significantly lower levels in cases compared to controls. In the three studies that examined manganese in hair, they all reported higher levels of manganese in cases compared to controls, and the two that examined manganese in nails reported significantly higher levels in cases compared to controls.

Lead in blood samples was examined by five studies, with three (60%) reporting higher levels in cases compared to controls; all three were statistically significant, and the other two (40%) showed lower levels in cases compared to controls and were also statistically significant. The one study that investigated lead concentrations in hair showed significantly higher levels in cases compared to controls, and the one study that examined lead in nails showed non-significant results.

Only three studies reported on arsenic in blood samples, and two (67%) of them reported significantly higher levels in cases compared to controls, and the other study, although it had lower levels in cases compared to controls, the results were not significant. Of the five studies that reported on nickel concentrations in blood samples, all five showed higher concentrations in the cases; four (80%) of them reported significantly higher concentrations in cases compared to controls, and the remaining one showed non-significant results. One study examined nickel in hair and nails, and it reported significantly higher levels of nickel in both samples. Only two studies reported on Hg, and only one (50%) had a higher and statistically significant level in cases compared to controls. One study investigated trace metals (Ni, Zn, Mn, and Se) in tissue. Zn and Se were significantly higher in controls compared to cases, while the levels for Mn were lower in controls, and they were not significant. However, for Ni, the levels were significantly lower in controls.

## 4. Discussion

The current data show a consistent pattern wherein males with prostate cancer had systemic and tissue depletion of supposedly beneficial micronutrients like zinc and selenium, together with higher loads of hazardous metals, most notably cadmium. In blood/serum, hair, nail, and tissue matrices, cadmium was the most reliably and robustly raised. In contrast, zinc and selenium demonstrated statistically significant decreases in cases compared to controls in several sample types. The differences for other non-essential or potentially genotoxic elements (lead, nickel, and mercury) were varied and generally smaller; some comparisons indicated slight increases in cases, which is in line with the metals’ high intra-group variance and low statistical power. Equivocal results were obtained for manganese with significant inter-study and inter-matrix heterogeneity. When selecting biomarkers for epidemiologic or clinical usage, consideration should be given to differential incorporation or exposure timing, as evidenced by repeatable intra-individual shifts found in paired sample comparisons (hair versus nail), especially for cadmium and zinc. While arsenic is a well-established carcinogen, the evidence linking it to PCa is weak. In this review, the studies measuring arsenic demonstrate slightly significant associations; this may demonstrate a lack of associations or variability in study populations. Hg, on the other hand, was slightly elevated in cases compared to controls, and evidence remained limited and inconsistent.

The main trend in these data has significant biological plausibility. Mechanistic studies show that cadmium can interact with androgen receptor pathways and modulate proliferative signals in prostatic epithelium [41,42]. Cadmium is a well-characterised environmental carcinogen with a long biological half-life, demonstrated ability to induce oxidative stress, interfere with DNA repair, and disrupt endocrine signalling [43,44,45]. Cadmium also exhibits estrogenic activity by binding to and activating oestrogen receptors; oestrogen signalling has been implicated in disease initiation and progression in the context of PCa [41]. Therefore, elevated systemic cadmium offers a plausible etiologic pathway to prostate cancer, particularly when paired with deficiencies in zinc and selenium [15,16,31,46]. Because zinc is essential for several DNA-repair enzymes and for normal prostate physiology (including citrate metabolism and redox balance), decreases in systemic or tissue zinc may compromise antioxidant defences and genomic integrity [47,48,49]. Glutathione peroxidases and thioredoxin reductases depend on selenium; its deficiency is predicted to reduce the body’s ability to neutralise reactive oxygen species and to make cells more susceptible to oxidative DNA damage [50,51,52]. A biologically feasible environment for the onset and progression of malignant transformation is defined by the combination of increasing exposure to toxicants and diminished antioxidant capacity.

The Newcastle–Ottawa Scale (NOS) was used to systematically evaluate the methodological quality of the included research, which is one of the review’s strengths. Accordingly, nearly all studies were assessed as good quality (NOS 7–9). Just one study had a middling quality rating (NOS = 6), and it was noteworthy that this study found fewer or non-significant correlations than the high-quality studies. This implies that the observed results might have been impacted by methodological rigour, and that the review’s conclusions might have been even more compelling if only high-calibre studies had been included.

It is important to exercise caution when interpreting the mixed results for arsenic, lead, nickel, and mercury. Although each of these metals has the potential to cause genotoxicity or endocrine disruption on its own [11,53,54], different exposure sources (such as occupational, food, smoking, and environmental contamination) affect the systemic concentrations of these metals [55,56,57,58,59]. The observed non-significant increases in lead or nickel may be the result of Type II error, actual but mild effects, or variations in exposure patterns across the populations examined. Elevated in a subset but not overall, mercury findings may also reflect subpopulations with occupational or dietary exposure [fish]; to improve mechanistic conclusions, speciation data [organic vs. inorganic mercury] would be needed.

The idea that metal dysregulation may play a role in prostate cancer is supported by the pattern of decreased levels of beneficial micronutrients like zinc and selenium and increased levels of harmful metals, especially cadmium. Mechanistically, this imbalance may promote the initiation and spread of tumours by aggravating oxidative stress, disrupting DNA repair pathways, and changing hormone receptor signalling. These results are consistent with earlier research that linked zinc deficiency and prolonged cadmium exposure to prostate cancer.

Along with micronutrient replacement techniques like supplementing zinc or selenium, cadmium depletion has been recommended as a possible way to lower the risk of prostate cancer or halt its progression [43]. Reducing cadmium exposure or body load may be a further preventive measure, given the documented estrogenic and genotoxic effects of cadmium and its constant escalation in cases. Before suggestions can be made, more clinical and mechanistic research is required, as the evidence for cadmium depletion is still early.

These findings have encouraging clinical and public health implications, but careful interpretation is needed. Low zinc/selenium status and increased cadmium load could be parts of a multi-element biomarker panel to stratify risk or to select people for focused prevention if they are reproduced in prospective cohorts. However, the randomised SELECT trial and other supplementation studies have produced mixed or null outcomes regarding selenium and vitamin-based prevention of prostate cancer, suggesting that simple repletion may not recapitulate physiologic protection and could carry unforeseen risks. As a result, clinical intervention (e.g., supplementation) should not be recommended based solely on cross-sectional associations.

## 5. Limitations

The current body of work in the subject matter has notable limitations that influence the validity and generalizability of these findings. The main concern is the varying methodologies across the studies involved, resulting in inconsistencies in confounding variables and contrasting results based on region or population. This gap has the potential to obscure the nuanced nature of trace metal exposure and its interaction with other lifestyle factors. The potential for confounding variables to skew results further complicates interpretations, as variations in sociodemographic factors, lifestyle, and genetic predisposition can influence both incidence and exposure. The fact that this evaluation solely includes case-control studies is one of its limitations. These investigations are limited in their ability to prove causality or timing, even if they offer direct comparisons of trace metal levels between controls and patients with prostate cancer. The exclusion of research written in languages other than English, which could introduce linguistic bias, is a limitation of this review. Furthermore, no search was conducted for grey literature (such as theses, dissertations, and conference proceedings). The one study that was not of high quality (NOS = 6) may have influenced the variability.

Additionally, many observational studies are subject to biases, particularly case-control studies, as participants may have selective recall of relevant behaviours or exposures. While current research provides crucial insights into the link between trace metals and PCa risk, these limitations underscore the necessity for a more standardised and comprehensive methodological approach to better elucidate this complex relationship.

## 6. Conclusions

To sum up, the current data support a model wherein elevated exposure to toxic metals, especially cadmium, combines deficiencies in protective micronutrients like zinc and selenium to produce a prostatic environment that is oxidatively stressed and DNA-repair compromised, which is favourable for the development of cancer. For cadmium and zinc/selenium, the results are robust across many matrices, biologically plausible, and generally compatible with the previous literature; nonetheless, they are still susceptible to confounding, reverse causation, and analytical heterogeneity. To test the observed associations and guide evidence-based prevention strategies, future research should provide priority to prospective designs with pre-diagnostic samples, standardised analytic methods, thorough confounder adjustment, speciation analyses when applicable, and mechanistic experiments.

## Figures and Tables

**Table 1:** Risk of bias assessment using the Newcastle–Ottawa Scale (NOS).

Study	Selection (Max 4 Stars)				Comparability (Max 2 Stars)	Outcome (Max 3 Stars)			Total Score (9 Stars)
	Adequate Case Definition?	Representativeness of Cases	Selection of Controls	Definition of Controls	Design or Analysis	Ascertainment of Exposure	Same Method of Ascertainment for Cases and Controls	Non-Response Rate	
[Bede-Ojimadu et al., 2023] [16]	*	*	*	*	**	*	*	*	9
[Lim et al., 2019] [17]	*	*	*	*	**	*	*	*	9
[Saleh et al., 2017] [18]	*	*	*	*	**	*	*	*	9
[Saleh et al., 2020] [19]	*	*	*	*	**	0	*	*	8
[Adedapo et al., 2012] [23]	*	*	*	*	**	*	*	*	9
[Amadi & Aleme, 2019] [24]	*	*	*	*	**	0	*	*	8
[Olooto et al., 2021] [25]	*	*	*	*	*	*	*	*	8
[Onyema-iloh et al., 2014] [26]	*	*	*	*	0	0	*	*	6
[Aydin et al., 2006] [27]	*	*	*	*	*	*	*	*	8
[Eken et al., 2016] [28]	*	*	*	*	**	*	*	*	9
[Kaba et al., 2014] [29]	*	*	*	*	*	*	*	*	8
[Ozmen et al., 2006] [30]	*	*	*	*	*	*	*	*	8
[Saleh et al., 2019] [31]	*	*	*	*	**	*	*	*	9
[Guntupalli et al., 2007] [32]	*	*	*	*	*	*	*	*	8
[Vinceti et al., 2007] [33]	*	*	*	*	*	*	*	*	8
[Karimi et al., 2012] [34]	*	*	*	*	**	*	*	*	9
[Steinbrecher et al., 2010] [35]	*	*	*	*	**	*	*	*	9
[Drozdz-Afelt et al., 2024] [36]	*	*	*	*	**	*	*	*	9
[Dhillon et al., 2022] [37]	*	*	*	*	*	*	*	*	9
[Chang et al., 2018] [38]	*	*	*	*	**	*	*	*	9
[Zaichick & Zaichick, 2019] [39]	*	*	*	*	**	0	*	*	8
[Qayyum & Shah, 2014] [40]	*	*	*	*	**	*	*	*	9

* Study has both exposure and control group; ** study included age or other sociodemographic factors.

## Data Availability

All data sets associated with this publication are presented in this manuscript.

## Supplementary Materials

The following supporting information can be downloaded at: https://www.mdpi.com/article/10.3390/cancers18020236/s1, Supplementary S1: search databases.
