# Supplementary material for: Presence of Trace Metals in the Biological Samples of Prostate Cancer Patients: A Systematic Review of Case-Control Studies
Source: Cancers (Basel). 2026 Jan 13;18(2):236. doi: 10.3390/cancers18020236 (PMC12838532; doi:10.3390/cancers18020236)
Supplement: Supplementary file 1 [file cancers-18-00236-s001.zip › cancers-3878171-supplementary.pdf]

Supplementary S1- search databases

1. **MeSH Terms and Keywords Used**
   - **Prostate cancer:** “prostate cancer”, “prostatic carcinoma”
   - **Trace metals:** “trace metals”, “trace elements”, “heavy metals”, "Cadmium", "Arsenic", "Mercury", "Nickel", "Lead", "Manganese", "Zinc", "Selenium"
   - **Exposure/Measurement:** "Environmental Exposure", "Biological Samples", "Blood", "Serum", "Urine", "Hair", "Tissue"
   - **Study Design:** "Case-Control Studies", "Observational studies"

2. **Search Syntax History**
   - **PubMed**
     ("Prostate cancer" OR "Prostate carcinoma")
     AND ("Trace Elements" OR "Cadmium" OR "Zinc" OR "Selenium" OR "Arsenic" OR "Mercury" OR "Nickel" OR "Lead" OR "Manganese")
     AND ("Case-Control Studies" OR "Case control" OR "Observational")
   - **ScienceDirect**
     ("prostate cancer" OR "prostatic neoplasm")
     AND ("trace element" OR cadmium OR zinc OR selenium OR arsenic OR mercury OR nickel OR lead OR manganese)
     AND ("case control" OR observational)
3. **Databases and Libraries Searched**
   - PubMed (MEDLINE)
   - ScienceDirect
